# Early Second-Look Hysteroscopy: Prevention and Treatment of Intrauterine Post-surgical Adhesions

**DOI:** 10.3389/fsurg.2019.00050

**Published:** 2019-08-16

**Authors:** Lauren Sebbag, Marc Even, Stéphanie Fay, Iptissem Naoura, Aurélie Revaux, Marie Carbonnel, Paul Pirtea, Dominique de Ziegler, Jean-Marc Ayoubi

**Affiliations:** Department of Obstetrics, Gynecology and Reproductive Sciences, Foch Hospital, Suresnes, France

**Keywords:** second look hysteroscopy, office hysteroscopy, adhesion prevention, intra uterine synechiae, intra-uterine adhesions, hysteroscopic resection

## Abstract

**Introduction:** Intra-uterine adhesion (IUA) is one of the main causes of secondary infertility. The aim of this study was to evaluate the prevalence of IUA developing in women undergoing hysteroscopic resection for submucous myomas, polyps, and intrauterine synechiae and test the efficacy of second look hysteroscopy for diagnosing and treating post-surgical adhesions.

**Materials and Methods:** We retrospectively collected data from reproductive age women who had a second look office hysteroscopy following hysteroscopic resection for myoma, polyp, or IUA at Foch hospital (Suresnes, France) between 2009 and 2017.

**Results:** Six hundred and twenty two reproductive-age women underwent hysteroscopic resection for myoma, polyp, and/or IUA. Among them, 155 women had a second look hysteroscopy. In this group, 29/155 (18.7%) had IUA formation: 17/83 (20.5%) women who underwent hysteroscopic myomectomy, 5/46 (10.9%) women who underwent hysteroscopic polypectomy, and 7/26 (26.9%) women who underwent hysteroscopic lysis of adhesions. These IUA have been lysed by the office hysteroscopy procedure in 16/29 (55.2%) patients: 11/17 (64.7%), 2/5 (40%), and 3/7 (42.9%) in women who underwent hysteroscopic myomectomy, polypectomy and lysis of adhesion, respectively.

**Conclusion:** IUA is a common complication of hysteroscopic surgery. Second look office hysteroscopy is an easy and effective procedure for diagnosing and removing newly formed IUA. It should be recommended for all women undergoing hysteroscopic resection for myomas, polyps, or IUA.

## Introduction

Intra-uterine adhesion (IUA) is an inflammatory reaction linking the opposed uterine walls. The resulting obliteration of the uterine cavity can be partial or total (Asherman's syndrome) (1). It most commonly occurs after traumas to the *basalis layer* of the endometrium most frequently, after curettage, cesarean sections, post-partum hemorrhage, abdominal myomectomy, and/or hysteroscopic resections of myoma, polyp, or uterine septum (2, 3). More rarely, it may also occur secondarily to infection (such as genital tuberculosis, endometritis).

IUAs can be responsible of symptoms including, chronic abdominal pain, menstrual abnormalities, and/or cause obstetric consequences like miscarriages, premature rupture of membranes (PROM), premature delivery, or placenta accreta (2). Furthermore, IUA is one of the main cause of secondary infertility with an incidence of 1.7–7% seen when hysteroscopies are performed in infertile women (4). We also know that hysteroscopic lysis of adhesion improves birth rate (32–46%) (5–10). Currently, hysteroscopy is the gold standard for the diagnosis and treating IUA (11). IUA can be classified in three stages, according to the American Fertility Society (AFS): mild (grade I), moderate (grade II), and severe (grade III) (12).

The purpose of the present study is to analyze prevalence of IUA development in women undergoing hysteroscopic resection for submucous myomas, polyps, and intrauterine synechiae and evaluate the efficiency of a second look hysteroscopy in order to diagnose and treat post-surgical adhesions.

## Materials and Methods

All patients undergoing surgery at our institution provided an informed consent authorizing the further analyses of their anonymized data, so Institutional Review Board approval was not necessary for this retrospective study.

In this retrospective cohort study, 622 reproductive-age women, between 18 and 45 years of age, underwent hysteroscopic resection for myoma, polyp, or IUA at our institution, Foch hospital (Suresnes, France) between January 2009 and March 2017. Among them, 155 patients had a second look office hysteroscopy following hysteroscopic surgery, 83 after myomectomy, 46 after polypectomy, and 26 after hysteroscopic lysis of adhesion for IUA. In the majority of cases (92/155), office hysteroscopy was performed as a second look procedure after an average time of 10.5 weeks. In the remaining cases (63/155) the post-surgical hysteroscopy was performed for other indications [infertility investigation, pre-IVF, atypical endometrial hyperplasia (AEH), dysfunctional uterine bleeding] with an average time interval of 47.4 weeks after the original surgery. A second look hysteroscopy was performed nearly systematically following hysteroscopic surgery starting from 2015. Data were collected from the operative and clinic records.

### Technique

The original hysteroscopic surgery was performed under general anesthesia during the first part of the menstrual cycle. A 30° forward-oblique resectoscope with an outer diameter of 9 mm was introduced and the resection was performed with monopolar (resectoscope Karl Storz, Tuttlingen, Germany) or bipolar energy (VersaPoint^®^ Gynecare, Somerville, NJ, USA) after prudent dilatation of the cervix by Hegar dilators. Physiologic saline (for bipolar resection) or glycine (for monopolar resection) was used to distend the uterine cavity. Antiadhesive gel (Hyalobarrier^®^) was freely used by surgeons. No antibiotics, hormonal therapy or IUD was used to prevent IUA.

The second look office hysteroscopy was performed without anesthesia. A 30° forward-oblique rectoscope (Bettocchi hysteroscope, Karl Storz^®^, Germany) with an outer diameter of 3 mm was used, without previous cervical dilatation. Physiologic saline or CO_2_ was used to distend the uterine cavity. The procedure allowed the diagnosis of post-surgical adhesions and also to perform adhesiolysis of newly formed mild IUA with the tip of the office hysteroscope. If recurrent IUA were too thick and had to be surgically excised repeat surgical hysteroscopy was planned.

## Results

The median age of women at the time of the surgery was 36.6 years. Mean gestation and parity were 1.3 and 0.4, respectively. In total, 81 (52.3%) patients had a past surgical history including, curettage for incomplete or elective abortion (14.8%), operative hysteroscopy for myoma (14.2%), polyp (5.2%) or IUA (1.3%), abdominal (7.1%) or laparoscopic (0.6%) myomectomy, and cesarean (5.2%). Ninety-five out of 155 patients (61.3%) suffered from infertility at the time of original surgery: 48 primary infertility (31%) and 47 secondary infertility (30.3%) (Table 1).

**Table 1:** Demographic characteristics.

**Characteristics**	**Patients (*n* = 155)**
Age (years)	36.6 ± 4.8
Weight (kg)	68.8 ± 16.2
Height (m)	1.65 ± 0.065
Body mass index (kg/m^2^)	25.4 ± 5.8
Gestity	1.3 ± 1.8
Parity	0.4 ± 0.9
Tobacco use (%)	4 (2.58%)
**Surgical history (%)**	81 (52.3%)
Curettage (%)	23 (14.8%)
Hysteroscopic polypectomy (%)	8 (5.2%)
Hysteroscopic myomectomy (%)	22 (14.2%)
Hysteroscopic adhesiolysis (%)	2 (1.3%)
Laparoscopic myomectomy (%)	1 (0.6%)
Abdominal myomectomy (%)	11 (7.1%)
Cesarean (%)	8 (5.2%)
**Infertility (%)**	95 (61.3%)
Primary infertility (%)	48 (31%)
Secondary infertility (%)	47 (30.3%)

Among the 155 patients, 7 had complications during the original surgery. Five patients had hydro-electrolytic disorders mainly hyponatremia due to the use of glycine, which resolved spontaneously and did not require intensive care. One patient experienced a post-operative hemorrhage with hypotension, tachychardia, and hemoglobin value of 6 g/dL, which motivated a laparoscopic exploration during which we found no hemoperitoneum but a vaginal wound caused by cervical dilatation, which was sutured. Another patient developed a per-operative bronchospasm and hypotension, which required intensive care treatment with orotracheal intubation, use of adrenaline and ephedrine. The bronchospasm resulted from an allergy to anesthetics products, which led to stop the surgical intervention. No cases of uterine perforation occurred. No complications happened during the second look hysteroscopy.

The postoperative office hysteroscopy revealed that 29 (18.7%) patients had IUA formations: 17/83 (20.5%) women who underwent hysteroscopic myomectomy, 5/46 (10.9%) women who underwent hysteroscopic polypectomy, and 7/26 (26.9%) women who underwent hysteroscopic adhesiolysis. These IUA have been lysed by the office hysteroscopy in 16/29 (55.2%) patients: 11/17 (64.7%), 2/5 (40%), and 3/7 (42.9%) women who underwent, respectively, hysteroscopic myomectomy, polypectomy, and adhesiolysis (Table 2).

**Table 2:** Office hysteroscopy results.

**Surgery indication**	**Patients**	**Hysteroscopic diagnostic of IUA**	**Hysteroscopic lysis of IUA**
Myoma	83 (53.5%)	17/83 (20.5%)	11/17 (64.7%)
Polyp	46 (29.7%)	5/46 (10.9%)	2/5 (40%)
Synechiae	26 (16.8%)	7/26 (26.9%)	3/7 (42.9%)
Total	155	29/155 (18.7%)	16/29 (55.2%)

*IUA, intra-uterine adhesion*.

## Discussion

The current study shows that IUA is a common finding following hysteroscopic surgery, with an incidence of 18.7% found at second look hysteroscopy. While IUA occurred most commonly after hysteroscopic lysis of adhesion (26.9%) and myomectomy (20.5%), they were also seen after hysteroscopic polypectomy (10.9%). We found that in 55.2% of cases, IUA could be treated by second-look hysteroscopy. These results therefore validate the need for performing a second look diagnostic hysteroscopy following surgical hysteroscopies.

Few studies have focused on second look office hysteroscopy as a preventive measure of post-operative IUA and only a small number of patients were included.

Pabuccu et al. (13) randomized 71 women who underwent hysteroscopic lysis of adhesion into 2 groups. Thirty-six patients in group 1 had a second-look hysteroscopy 1 week following surgery (with further IUA lysis) and a third-look hysteroscopy 2 months later. Thirty-five patients in group 2 had a second-look hysteroscopy 2 months later. Both groups had an intrauterine device (IUD) inserted during the original hysteroscopic lysis of adhesion and received 2 months of estrogen and progestin therapy. The IUA formation rate was significantly lower in Group 1: no adhesion was detected in 33 (89.1%) of 36 patients in group 1 and 6 (17.1%) of 35 patients in group 2 (*p* < 0.05). Globally, these data show that early second-look hysteroscopy improves the ultimate success of surgery.

Robinson et al. (14) retrospectively evaluated 24 patients treated with primary hysteroscopic lysis of adhesion followed by hormone therapy and serial flexible office hysteroscopy. They found that 92% (22/24) of patients had an overall improvement in the stage of their Asherman's syndrome.

Yang et al. (15) reported data on 153 women who had a hysteroscopic myomectomy for single or multiple (apposing or not) myoma. They were divided into 4 groups with different IUA prevention strategies. Diagnostic office hysteroscopy was done 1–3 months after surgery and revealed that postoperative adhesions are common in women who had apposing myomas (despite IUD) but were not found in any of the women (*n* = 7) undergoing office hysteroscopic early lysis.

The optimal interval for realizing the second look hysteroscopy has not been established yet but it is believed that early dissection during second look hysteroscopy has a positive outcome on the ultimate risk of developing new synechiae. Some authors recommend very early hysteroscopy (13, 15), however there is no solid evidence for such claim. One of the limitations of this study is the various time interval between the operative and the office hysteroscopy, but most of them were performed early after the surgery as a second look procedure.

According to Shokeir et al. (16) IUAs formed immediately after the surgery are histologically different from those appearing a longer time after the operation. Early occurring IUAs are mainly composed of grade I vs. grade II/III. Indeed, early office hysteroscopy allows the lysis of newly formed adhesions, which are thin and filmy, whereas delayed adhesions are thick and fibrous and need a surgical lysis of adhesion (14). In this study we unfortunately did not have data about adhesion types.

Office hysteroscopy may be a way of verifying the effectiveness of preventive anti-adhesives. Various measures of preventing adhesions were studied. Anti-adhesive gels, such as hyaluronic acid gel (Hyalobarrier^®^) are the most widely used and have a significant clinical effect on IUA prevention (17–19). However, their effect on further pregnancy rate is unknown. These results must be confirmed by prospective randomized trials before we can recommend their general use. The role of intrauterine device (IUD), hormonal and antibiotic therapy are difficult to evaluate because generally, these have been used in association with other prevention strategies (4). Only one low-power randomized trial studied the benefit of the intrauterine device (IUD) with or without estrogen treatment to prevent IUA after operative hysteroscopy but no significant result was found (20). Neither technique can be recommended for routine use (11). Finally, bipolar resection seems to be associated with a lower IUA recurrence rate compared to monopolar resection, but randomized prospective studies are still needed to confirm this result (21).

Regarding reproductive outcomes, no study has demonstrated its effectiveness in improving spontaneous fertility but it seems that control hysteroscopy may improve pregnancy rates: 47 vs. 30% (13).

The main limitation of this study is its retrospective nature. Followed by the small number of patients who underwent second look hysteroscopy. Unfortunately due to the retrospective design of the study, we were not able to collect subsequent fertility data, which could have made the study more interesting. Therefore high-quality randomized studies are still needed to strongly recommend systematic office hysteroscopy after hysteroscopic resection for submucosal myomas, polyps, or synechiae.

Second look office hysteroscopy is shown to be a simple, safe and useful procedure for investigating intrauterine lesions. It allows identification of post-surgical adhesions and early treatment before they become thick and a new intervention is needed.

## Conclusion

In conclusion, second look office hysteroscopy is an easy and effective procedure for diagnosing and removing newly formed IUA. Second look hysteroscopy could prevent the development of moderate or IUA and therefore improve reproductive outcome. Routine early second-look hysteroscopy should be recommended for all women undergoing hysteroscopic resection for myomas, polyps, or synechiae within 6 weeks after the surgery.

## Data Availability

The raw data supporting the conclusions of this manuscript will be made available by the authors, without undue reservation, to any qualified researcher.

## Ethics Statement

All patients undergoing surgery at our institution provided an informed consent authorizing the further analysis of their anonymized data, so Institutional Review Board approval and written consent was not necessary for this retrospective study in accordance with the local legislation and institutional requirements. According to the French Public Health Code (article L1123-7-25th May 2018), only research which involves directly the patient participation requires an approval from an IRB/IEC. In the case of our research, only data collection from medical records has been performed. This kind of research does not involve directly the patient participation, does not requires ethical review or written consent.

## Author Contributions

LS and ME contributed to the protocol and project development, data collection and management, data analysis, and manuscript writing and editing. SF, IN, AR, MC, and PP contributed to the protocol and project development. DZ and J-MA contributed to the manuscript writing and editing.

### Conflict of Interest Statement

The authors declare that the research was conducted in the absence of any commercial or financial relationships that could be construed as a potential conflict of interest.
